# Supplementary material for: Electro-assisted methane oxidation to formic acid via in-situ cathodically generated H2O2 under ambient conditions
Source: Nat Commun. 2023 Aug 5;14:4704. doi: 10.1038/s41467-023-40415-6 (PMC10404228; doi:10.1038/s41467-023-40415-6)
Supplement: Supplementary file 1 — Supplementary Information [file 41467_2023_40415_MOESM1_ESM.pdf]

Supplementary Information

Electro-assisted methane oxidation to formic acid via *in-situ* cathodically generated H_2O_2 under ambient conditions

Kim et al.

Supplementary Figures

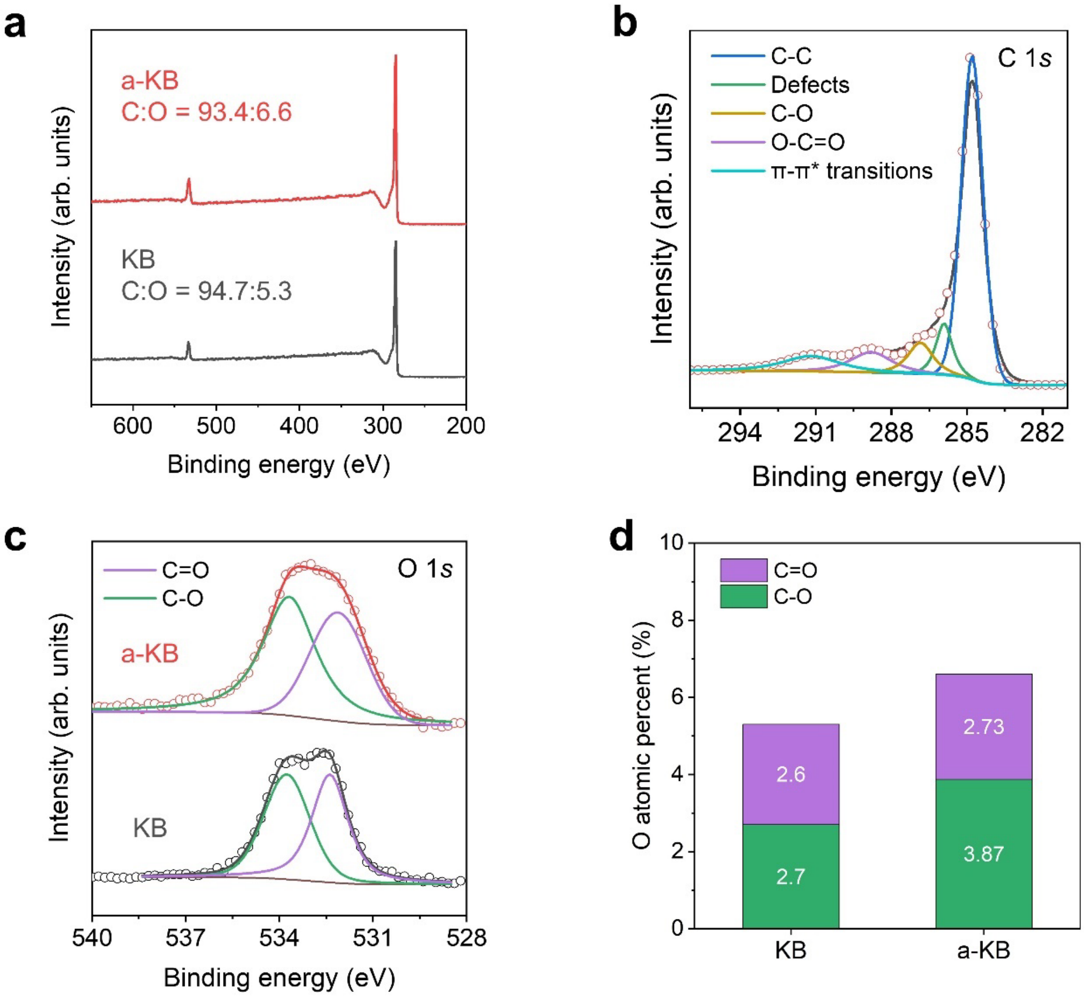

Supplementary Figure 1. (a) XPS survey spectra of KB and a-KB. (b) C 1*s* spectrum of a-KB. (c) O 1*s* spectrum of KB and a-KB. (d) The content of C-O and C=O functional groups in KB and a-KB. Source data are provided as a Source Data file.

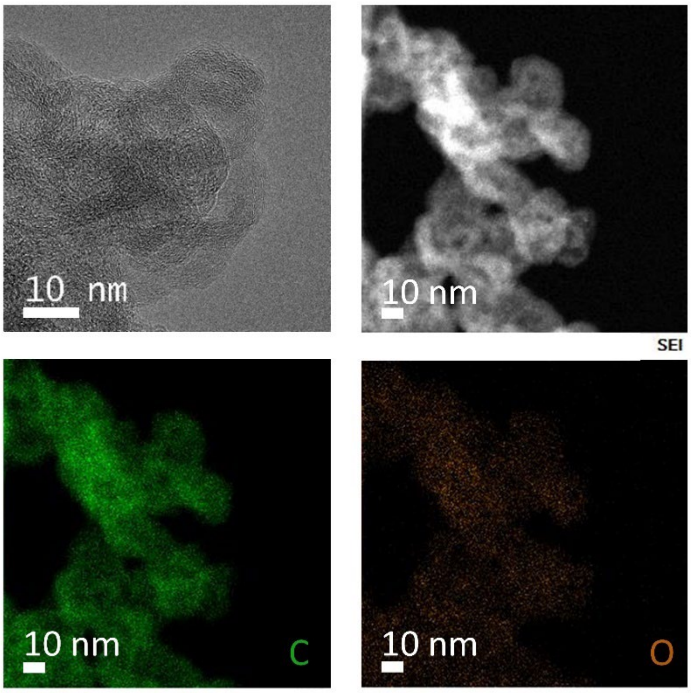

Supplementary Figure 2. HR-TEM and EDS mapping images of a-KB.

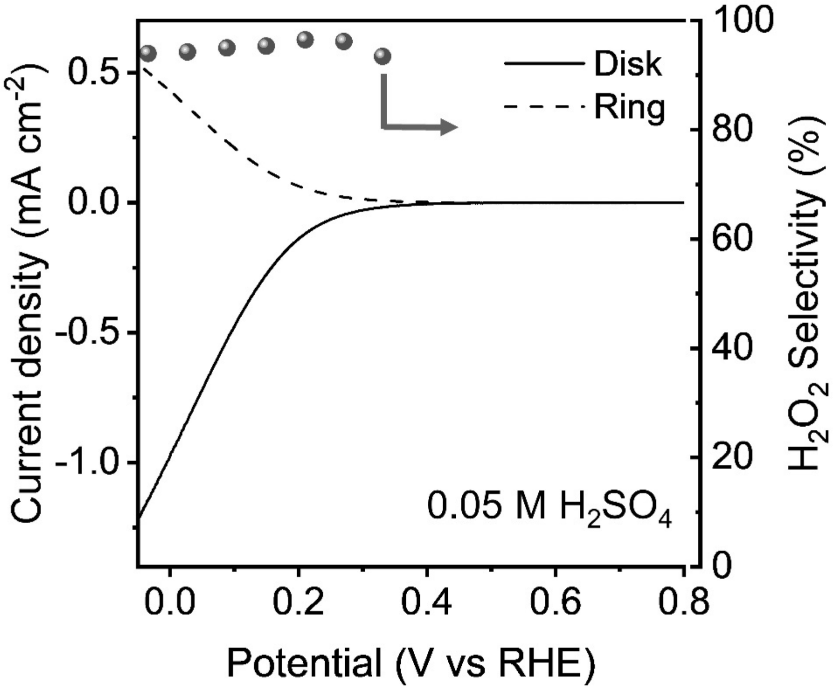

Supplementary Figure 3. Electrochemical ORR performance for H_2O_2 production of a-KB in an acidic electrolyte (0.05 M H_2SO_4). Source data are provided as a Source Data file.

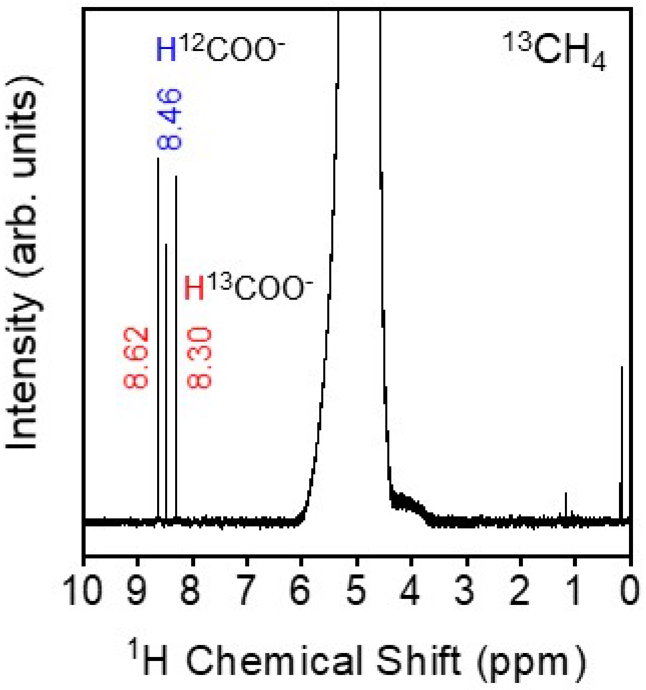

Supplementary Figure 4. ^{1}H-NMR analysis of EMPO products applying $^{13}CH4$. The peaks at 8.30, 8.62 are attributed to $H^{13}COO^{-}$ and 8.46 ppm is for $H^{12}COO^{-}$, respectively. Source data are provided as a Source Data file.

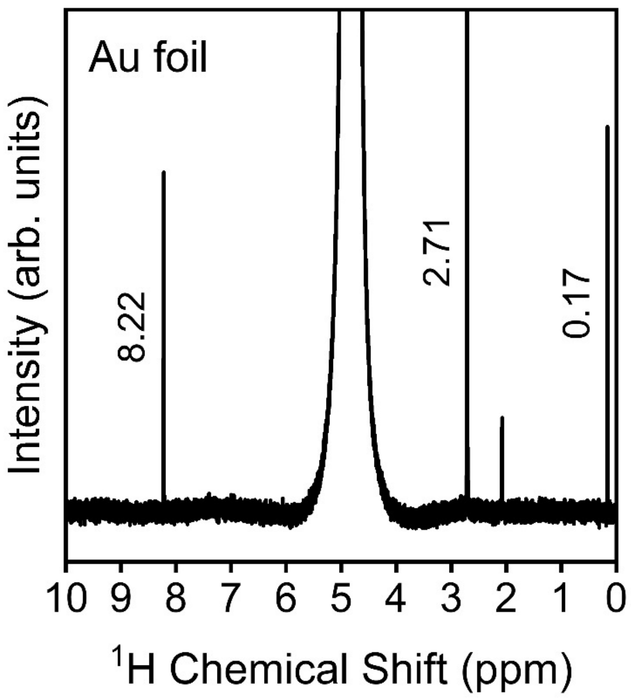

Supplementary Figure 5. ^{1}H-NMR analysis after EMPO reaction applying Au foil as a carbon-free ORR catalyst. The peaks at 0.17, 2.71, and 8.22 ppm are attributed to CH_4, $(CH_3)_2SO$ (DMSO), and HCOOH, respectively. Source data are provided as a Source Data file.

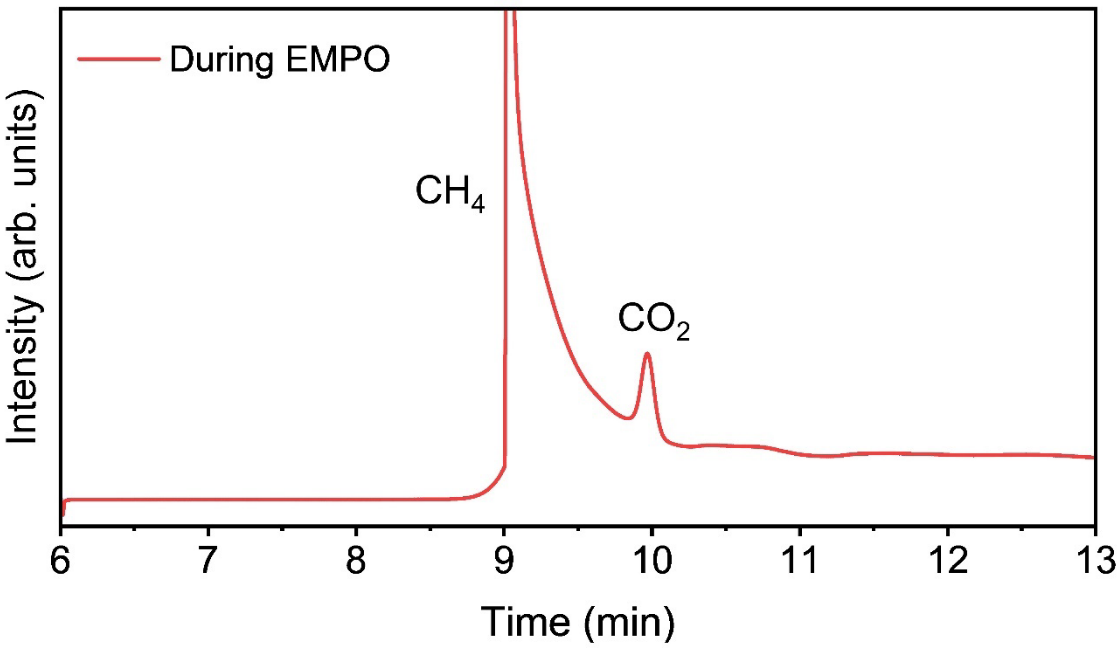

Supplementary Figure 6. The FID Gas chromatogram during EMPO reaction. Source data are provided as a Source Data file.

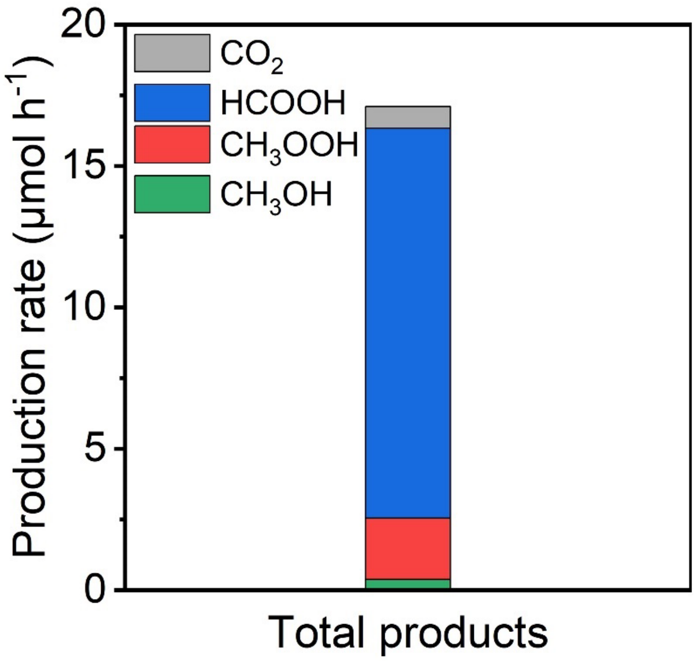

Supplementary Figure 7. Production rate of total products. Reaction conditions: 0 V (vs. RHE), 25 °C, 1 bar, 30 min, O_2: 100 sccm, CH_4: 100 sccm, 55 mL of 0.05 M H_2SO_4, and stirring at 700 rpm. Source data are provided as a Source Data file.

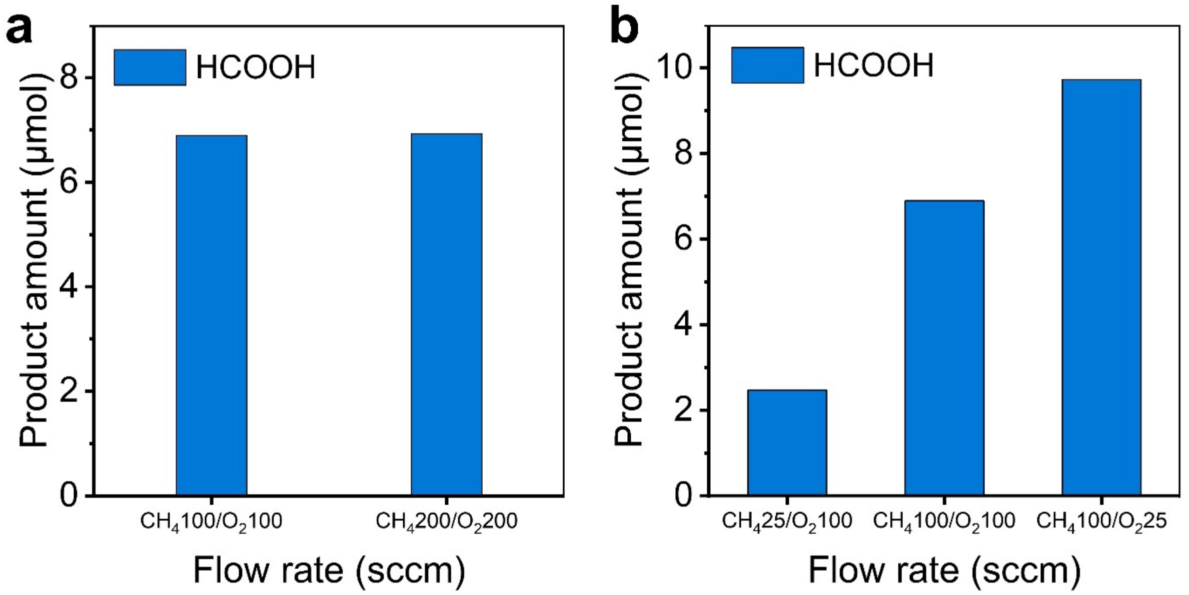

Supplementary Figure 8. The product amount of HCOOH depending on the (a) total flow rate and (b) flow rate ratio of CH_4:O_2. Reaction conditions: 0 V (vs. RHE), 25 °C, 1 bar, 30 min, O_2: 100 sccm, CH_4: 100 sccm, 55 mL of 0.05 M H_2SO_4, and stirring at 700 rpm. Source data are provided as a Source Data file.

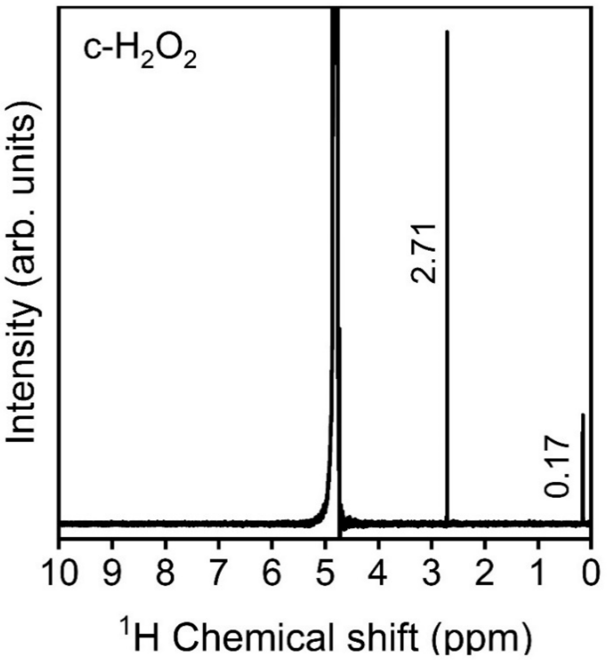

Supplementary Figure 9. ^{1}H-NMR spectrum of the products after reaction with CH_4 and c-H_2O_2 at 25 ^oC. The peaks at 0.17 and 2.71 ppm are attributed to CH_4 and $(CH_3)_2SO$ (DMSO), respectively. Source data are provided as a Source Data file.

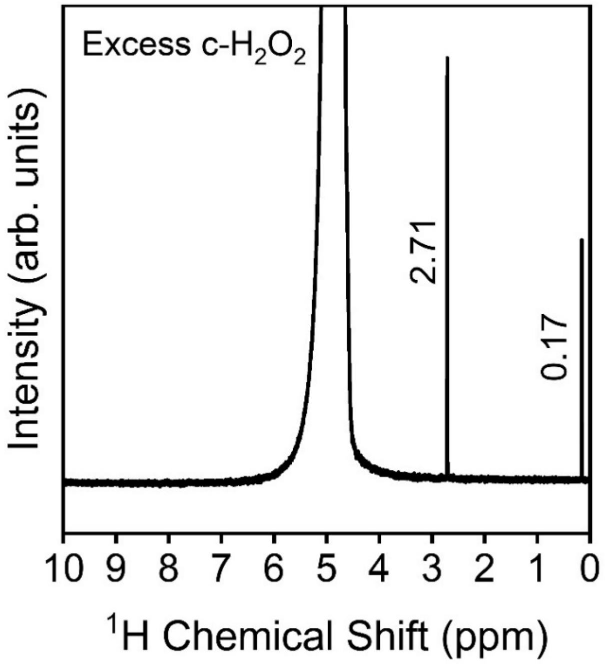

Supplementary Figure 10. ^{1}H NMR spectrum of the products after reaction with CH_4 and excess amount of c-H_2O_2 (10 times more than that of c-H_2O_2) at 25 °C. The peaks at 0.17 and 2.71 ppm are attributed to CH_4 and $(CH_3)_2SO$ (DMSO), respectively. Source data are provided as a Source Data file.

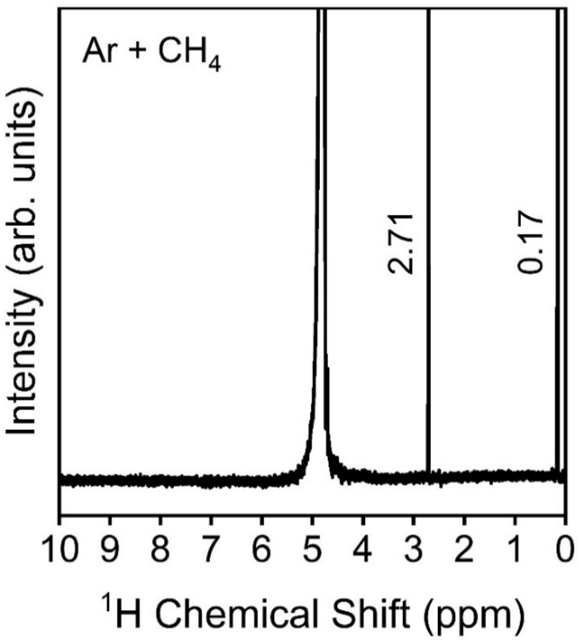

Supplementary Figure 11. ^{1}H-NMR analysis after EMPO reaction replacing O_2 with Ar. The peaks at 0.17 and 2.71 ppm are attributed to CH_4 and $(CH_3)_2SO$ (DMSO), respectively. Reaction conditions: 0 V (vs. RHE), 25 °C, 1 bar, 30 min, Ar: 100 sccm, CH_4: 100 sccm, 55 mL of 0.05 M H_2SO_4, and stirring at 700 rpm. Source data are provided as a Source Data file.

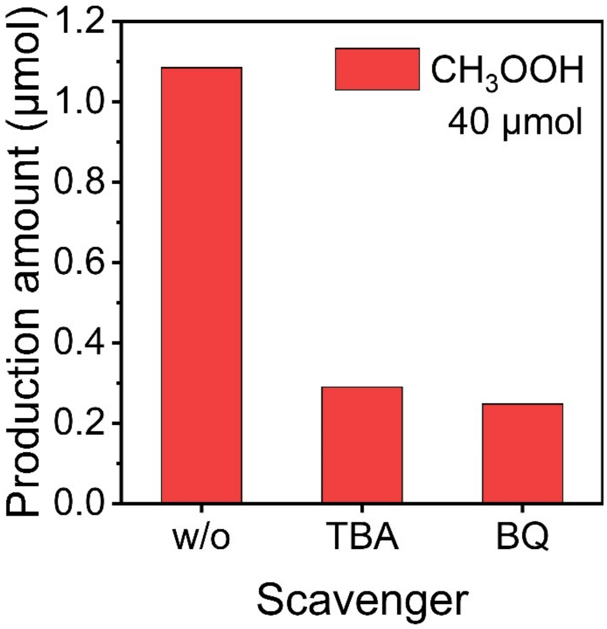

Supplementary Figure 12. CH_3OOH production after EMPO reaction in the presence of 40 μmol of scavengers. TBA and BQ are radical scavengers that can trap ·OH and ·OOH, respectively. Source data are provided as a Source Data file.

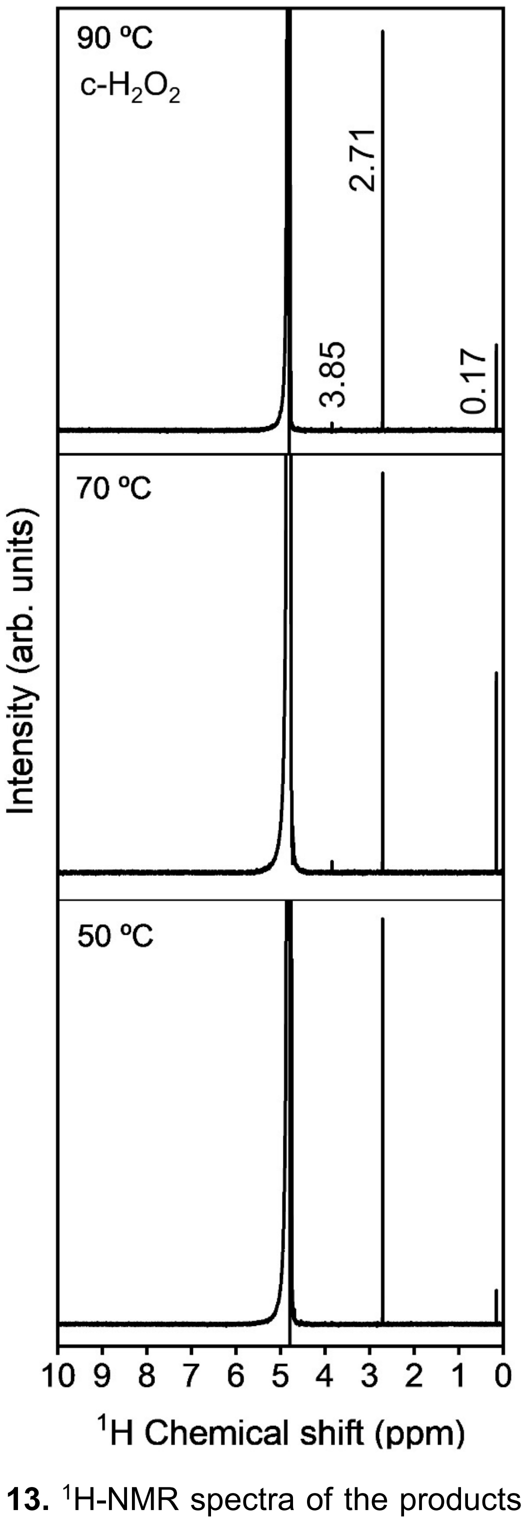

Supplementary Figure 13. ^{1}H-NMR spectra of the products after reaction with CH_4 and c-H_2O_2 at elevated temperatures (50-90 °C). The peaks at 0.17, 2.71, and 3.85 ppm are attributed to CH_4, $(CH_3)_2SO$ (DMSO), and CH_3OOH, respectively. Source data are provided as a Source Data file.

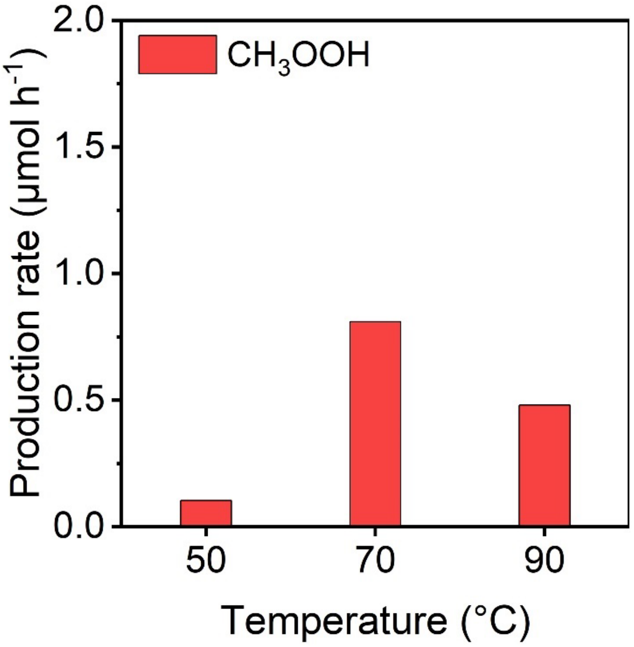

Supplementary Figure 14. CH_3OOH production rates after CH_4 oxidation reaction with commercial H_2O_2 at elevated temperatures (50-90 °C). Source data are provided as a Source Data file.

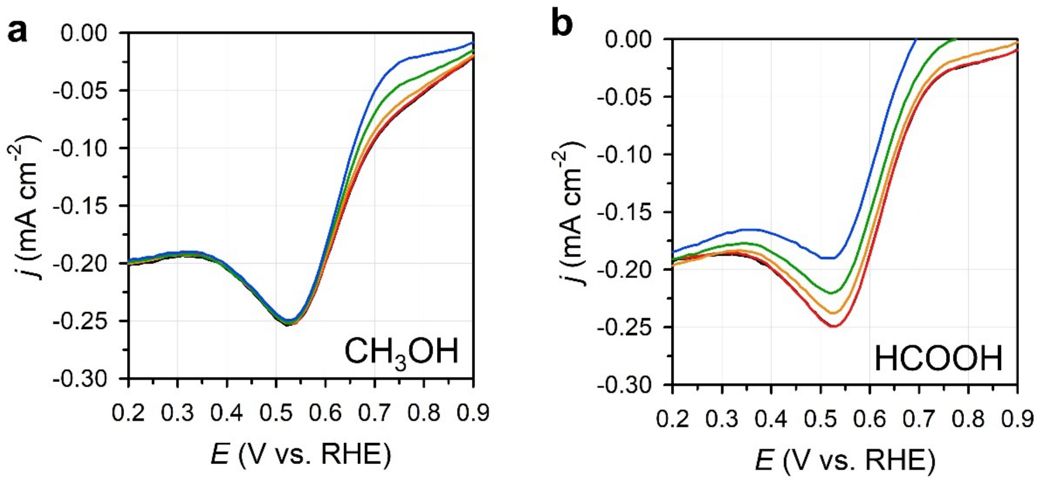

Supplementary Figure 15. The polarization curves in the presence of 0~40 mM (a) CH_3OH, and (b) HCOOH in 0.05 M H_2SO_4 electrolyte. The black, red, orange, green, and blue lines indicate blank, 5, 10, 20, and 40 mM, respectively. Source data are provided as a Source Data file.

In comparison with CH_3OOH, only capacitive currents were observed when various concentrations of CH_3OH and HCOOH were applied. The capacitive current is affected by double layer thickness. With increasing double layer thickness, the current density is decreased. The HCOOH may affect double layer thickness inducing decrease capacitive current upon increasing HCOOH concentration.

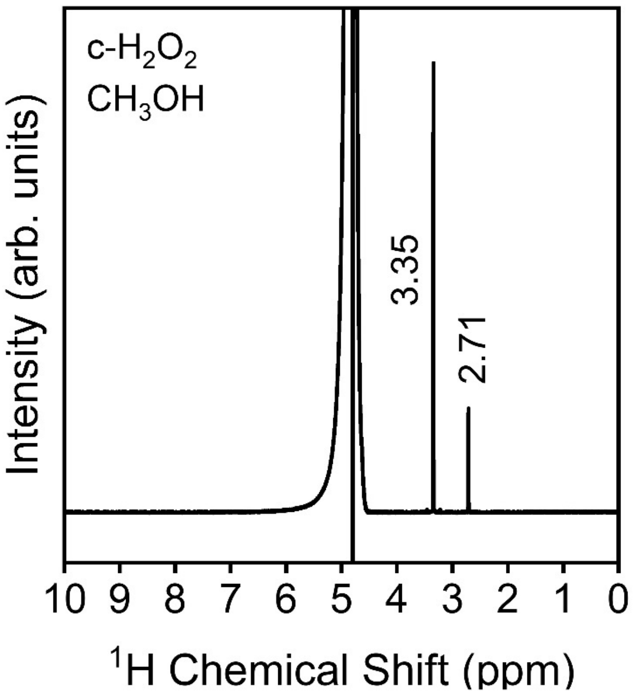

Supplementary Figure 16. ^{1}H-NMR spectrum of electrolyte after reaction between CH_3OH and c-H_2O_2 at 25 °C. The peaks at 2.71, and 3.35 ppm are attributed to $(CH_3)_2SO$ (DMSO), and CH_3OH, respectively. Source data are provided as a Source Data file.

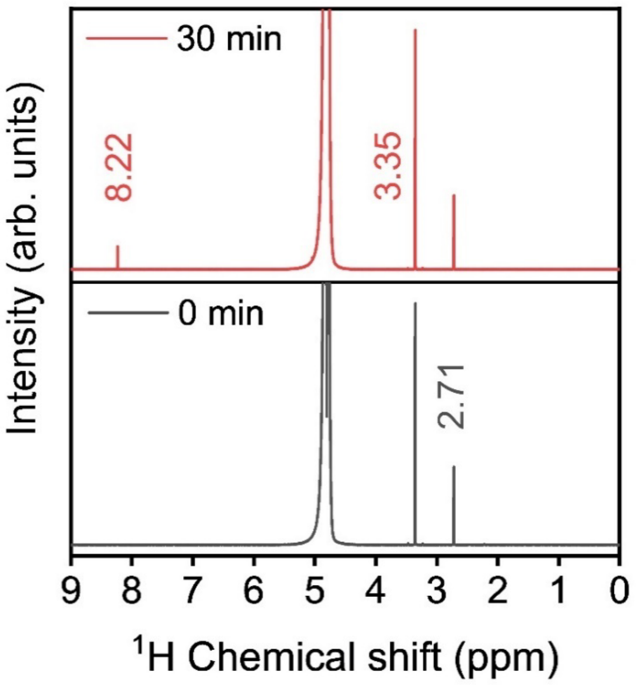

Supplementary Figure 17. ^{1}H-NMR analysis of methanol oxidation products using ORR at each reaction time. The peaks at 2.71, 3.35 and 8.22 ppm are attributed to $(CH_3)_2SO$ (DMSO), CH_3OH, and HCOOH, respectively. Source data are provided as a Source Data file.

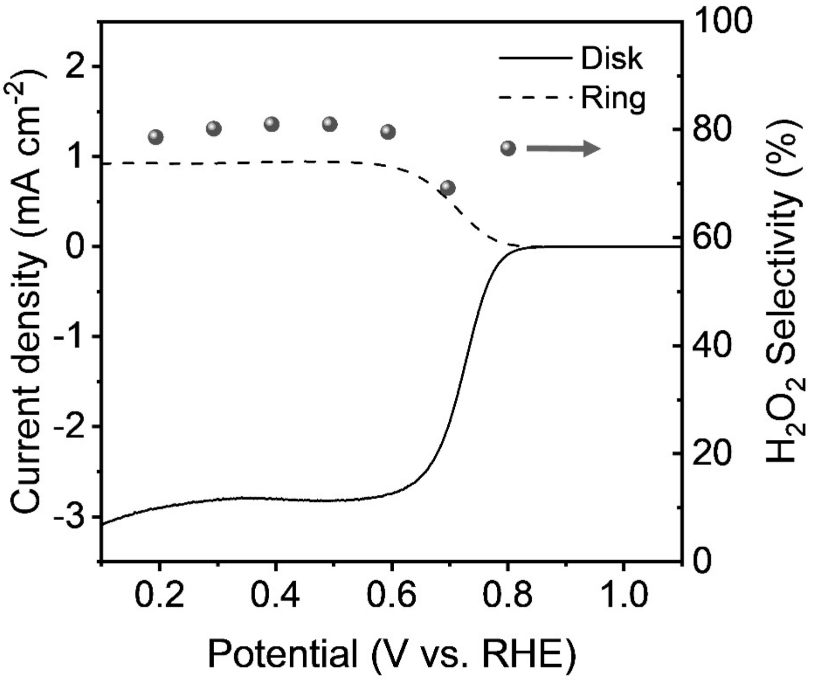

Supplementary Figure 18. Electrochemical ORR performance for H_2O_2 production of a-KB in an alkaline electrolyte (0.1 M KOH). Source data are provided as a Source Data file.

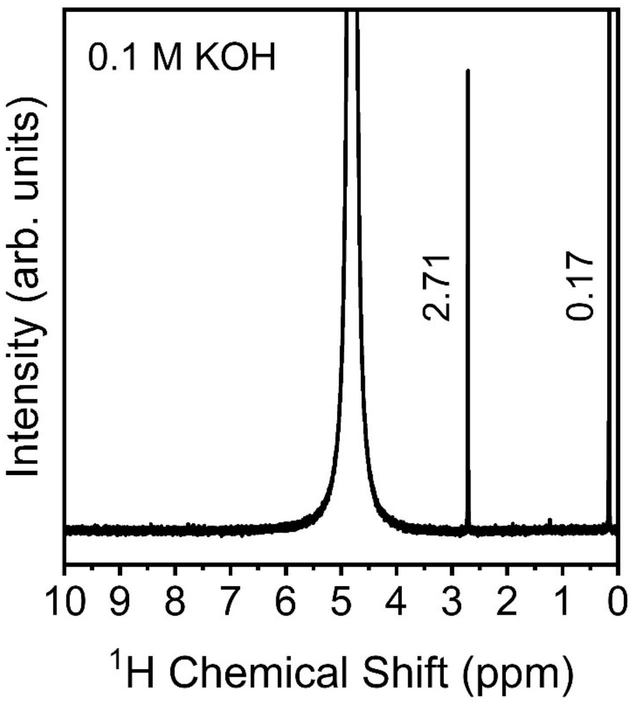

Supplementary Figure 19. ^{1}H-NMR spectrum of the products after EMPO reaction in the alkaline electrolyte. The peaks at 0.17 and 2.71 ppm are attributed to CH_4 and $(CH_3)_2SO$ (DMSO), respectively. Reaction conditions: 0 V (vs. RHE), 25 °C, 1 bar, 30 min, O_2: 100 sccm, CH_4: 100 sccm, 55 mL of 0.1 M KOH, and stirring at 700 rpm. Source data are provided as a Source Data file.

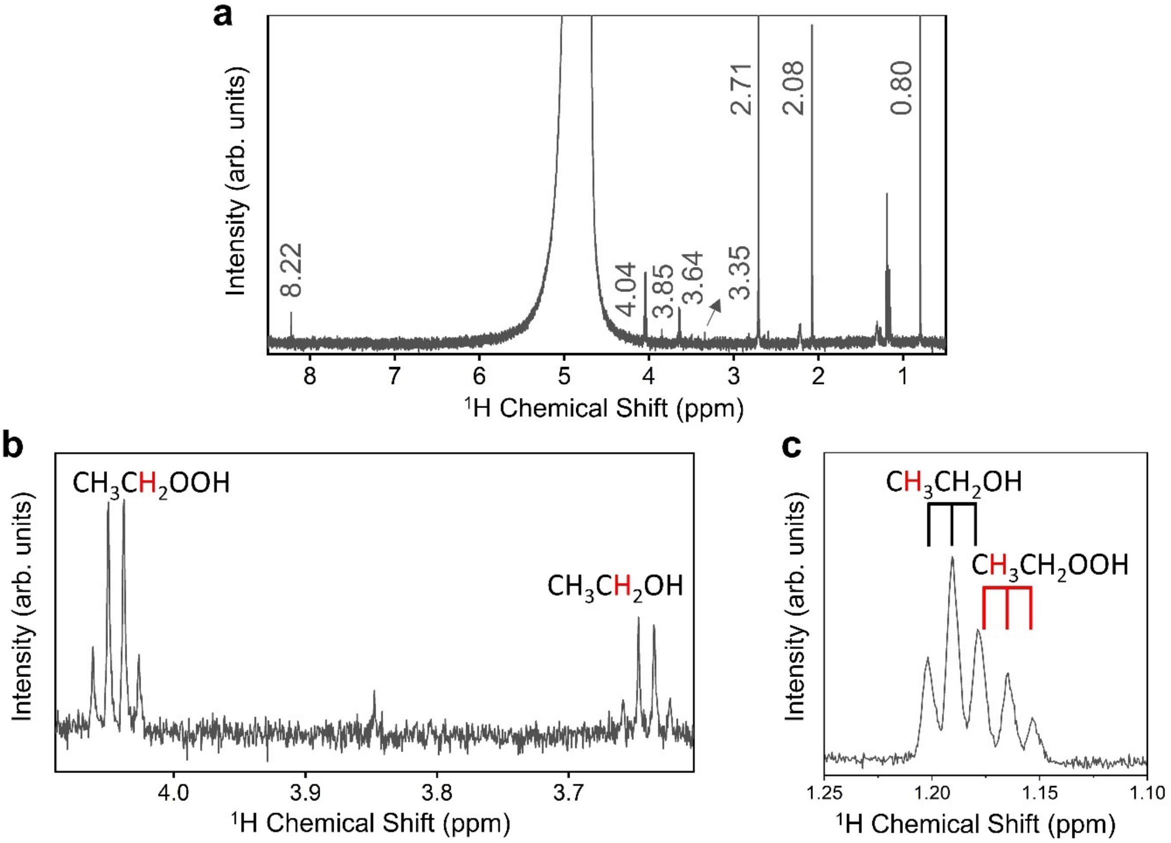

Supplementary Figure 20. (a) ^{1}H-NMR analysis of electro-assisted C_2H_6 partial oxidation products using ORR. The peaks at 0.80, 2.08, 2.71, 3.35, 3.64, 3.85, 4.04, and 8.22 ppm are attributed to C_2H_6, CH_3COOH, $(CH_3)_2SO$ (DMSO), CH_3OH, C_2H_5OH, CH_3OOH, C_2H_5OOH and HCOOH, respectively. Enlarged ^{1}H-NMR spectra (b) from 3.6 to 4.1 and (c) from 1.10 to 1.25 ppm chemical shift. Source data are provided as a Source Data file.

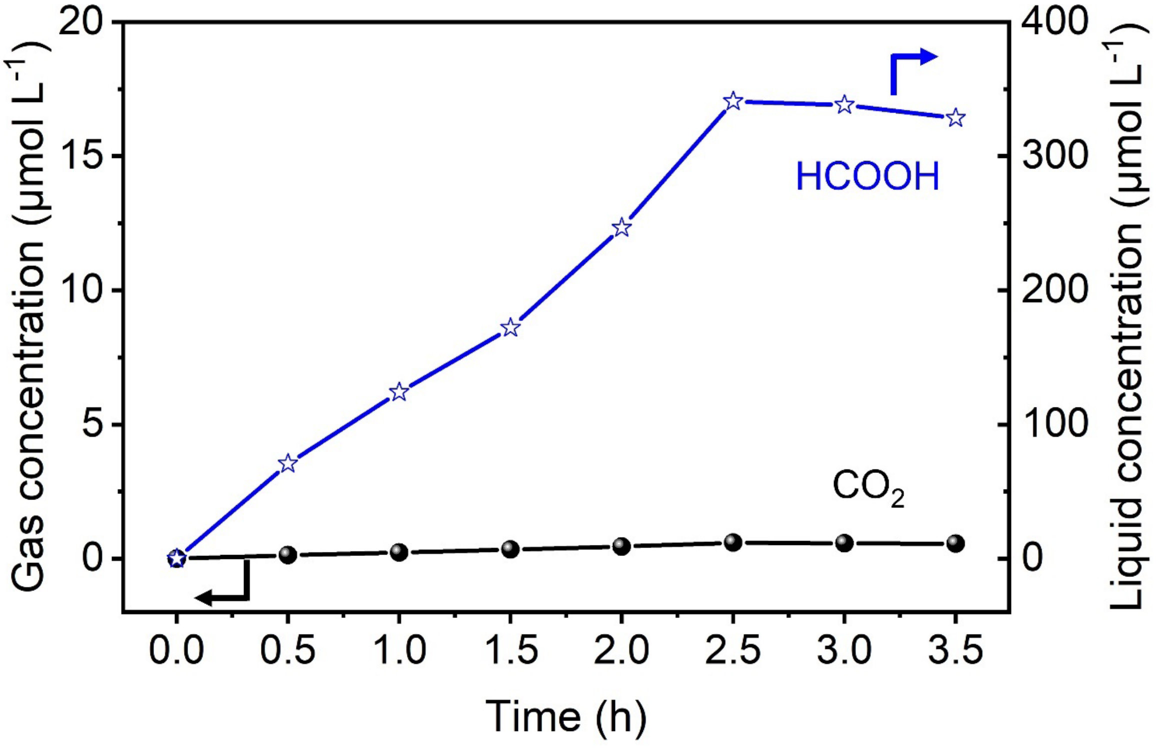

Supplementary Figure 21. Gas (CO_2) and liquid (HCOOH) concentration changes upon reaction time.

The concentration of CO_2 was significantly lower compared to HCOOH, and it was slightly increased as that of HCOOH was increased. Then CO_2 concentration was kept constant once the concentration of HCOOH reached saturated value confirming that increased concentration of HCOOH induces over-oxidation to CO_2. Source data are provided as a Source Data file.

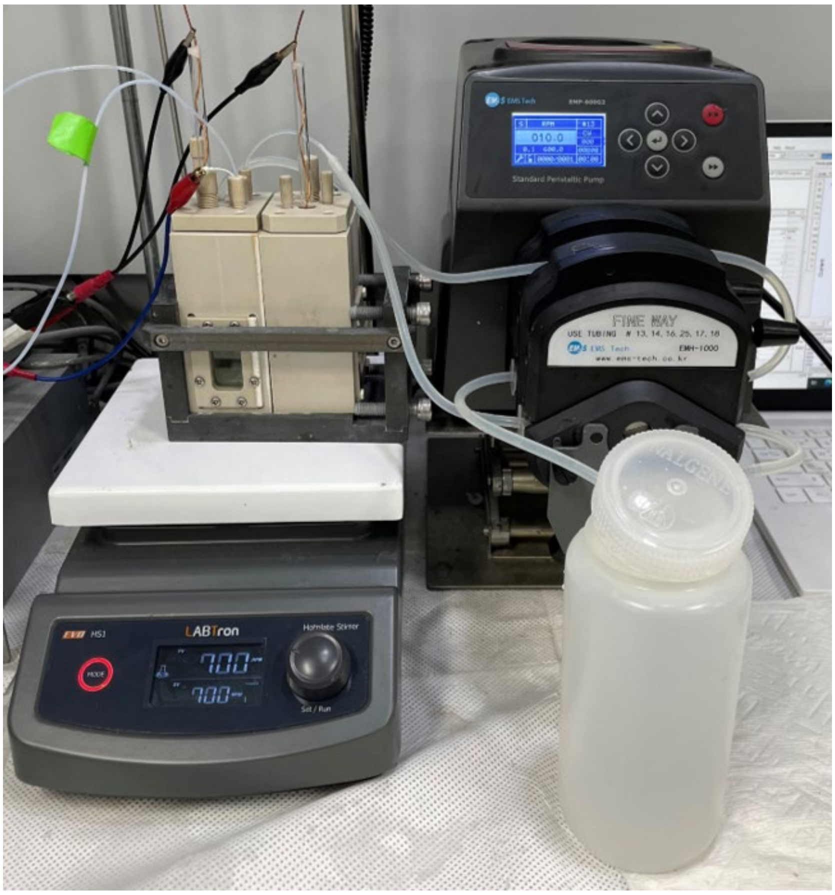

Supplementary Figure 22. The electrolyte-flowing H-cell setup for long-term operation experiment.

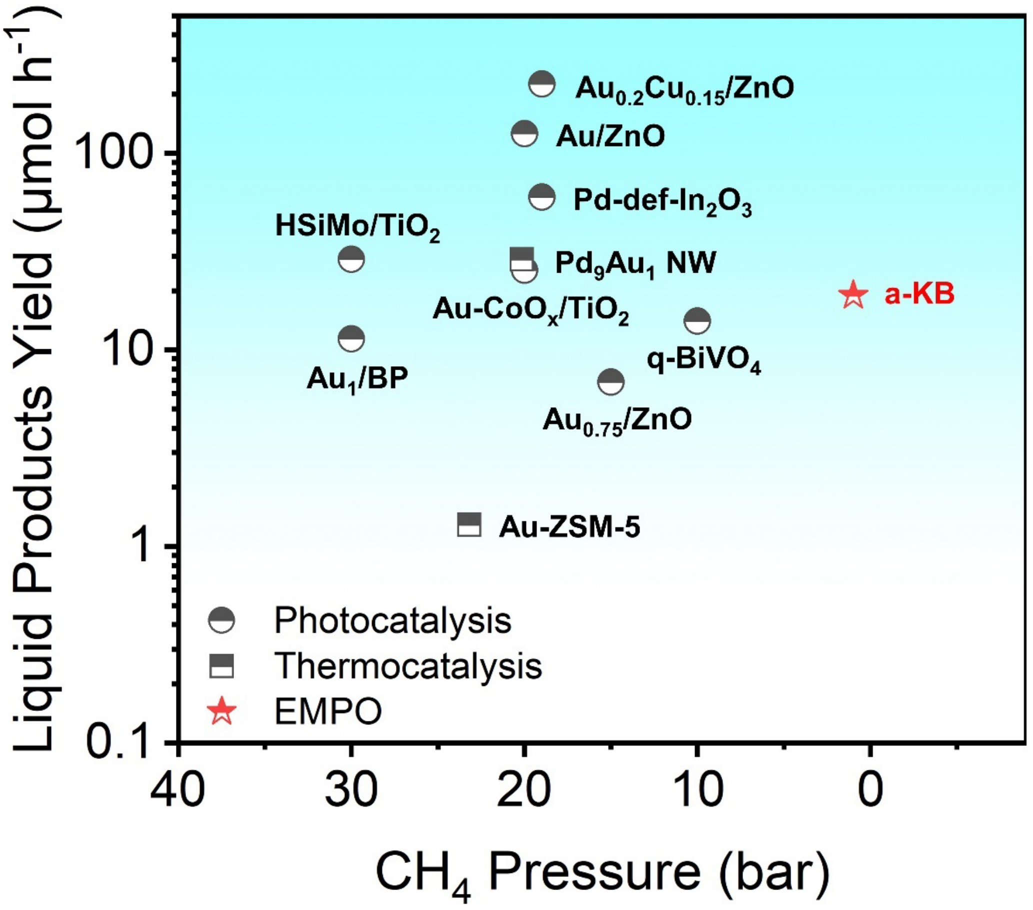

Supplementary Figure 23. Comparison of liquid products yield and CH_4 pressure with reported Thermo- and Photocatalysis, and EMPO system. Source data are provided as a Source Data file.

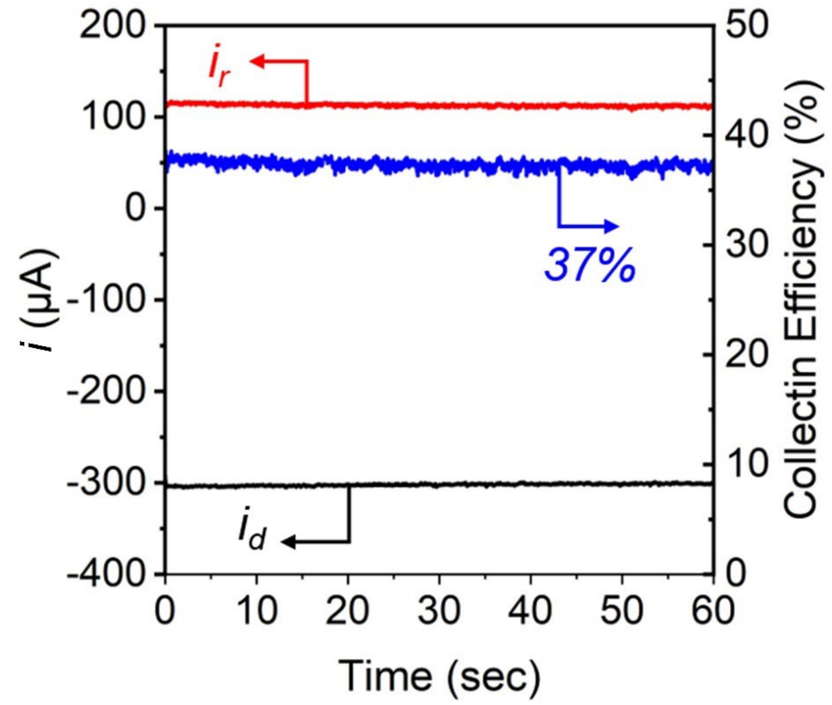

Supplementary Figure 24. Chronoamperometric response of the RRDE at -0.3 V (vs. Ag/AgCl) with an electrode rotation speed of 1600 rpm measured in 2 mM $K_3[Fe(CN)_6]$ + 0.1 M KOH electrolyte. Source data are provided as a Source Data file.

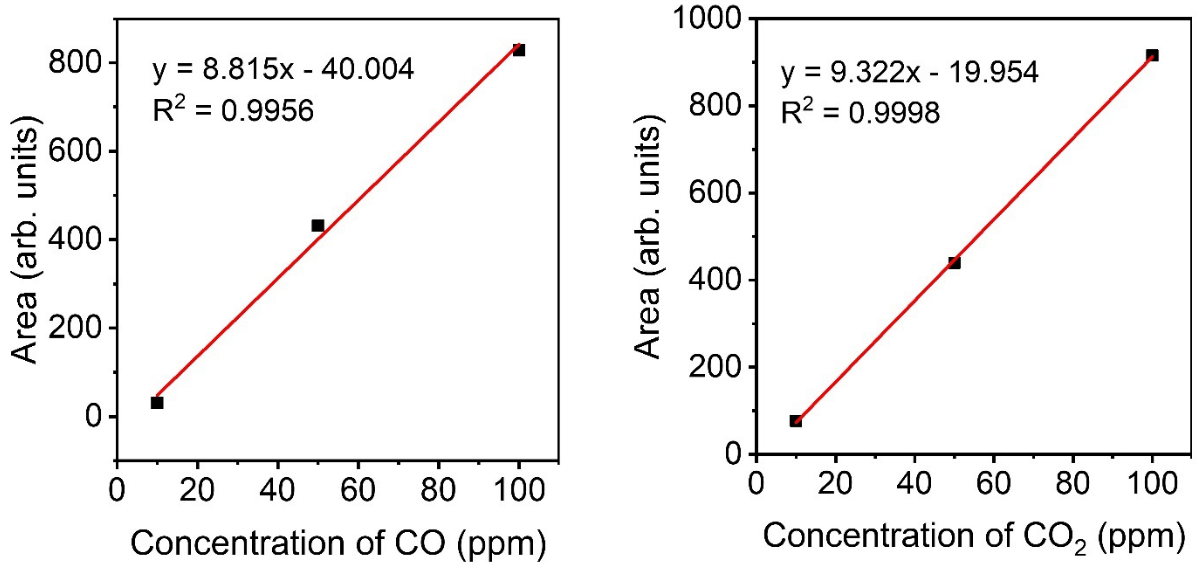

Supplementary Figure 25. Calibration curves of the GC for CO and CO_2 gases. Source data are provided as a Source Data file.

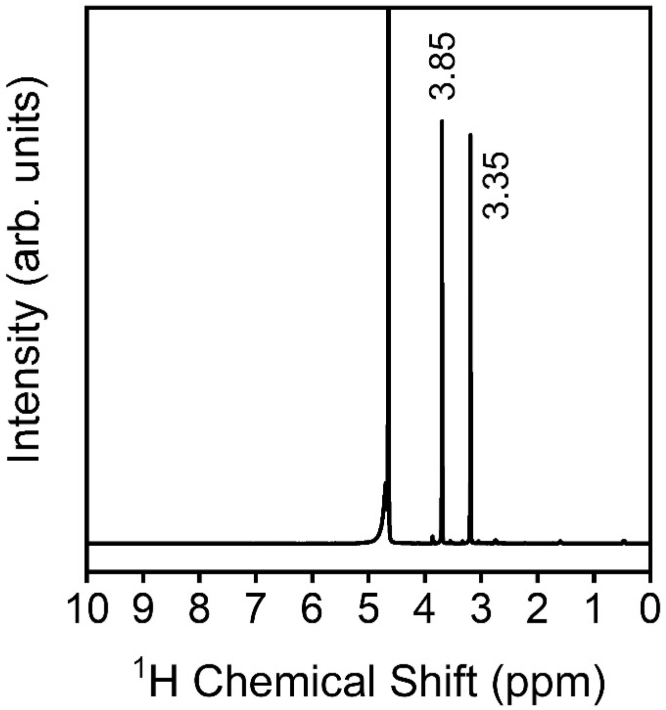

Supplementary Figure 26. ^{1}H-NMR spectrum of the synthesized CH_3OOH. The peaks at 3.84 and 3.33 ppm are attributed to CH_3OOH and CH_3OH, respectively. Source data are provided as a Source Data file.

Supplementary Tables

Samples	Concentration of Pt
a-KB catalyst (after)	0 ppm
Electrolyte (before)	0 ppm
Electrolyte (after)	0 ppm

Supplementary Table 1. The ICP-OES results of Pt concentration of an a-KB catalyst and electrolytes.

Catalyst	Reaction condition	Liquid products		Ref.
		Product	Selectivity (%)	
a-KB	EMPO<br>11.25 mg catalyst,<br>1 bar CH_4,<br>25 °C	Total: 18.9 μmol·h^{-1}<br>CH_3OH + CH_3OOH + HCOOH	CH_3OH: 2.1<br>CH_3OOH: 14.3<br>HCOOH: 83.6	This Work
Au/ZnO	Photocatalysis,<br>10 mg catalyst,<br>20 bar CH_4,<br>25 °C	Total: 125.5 μmol·h^{-1}<br>CH_3OH + CH_3OOH + HCHO	CH_3OH: 16.4<br>CH_3OOH: 49.2<br>HCHO: 34.4	*J. Am. Chem. Soc.* **2019**, 141, 20507-20515.
$Au_{0.75}$/ZnO	Photocatalysis,<br>10 mg catalyst,<br>15 bar CH_4<br>30 °C	Total: 6.9 μmol·h^{-1}<br>CH_3OH	CH_3OH: 100	*J. Mater. Chem. A* **2020**, 8, 13277-13284
Au-CoO_x/TiO_2	Photocatalysis,<br>10 mg catalyst,<br>20 bar CH_4,<br>25 °C	Total: 25.4 μmol·h^{-1}<br>CH_3OH + CH_3OOH	CH_3OH: 56.7<br>CH_3OOH: 43.3	*ACS Catal.* **2020**, 10, 14318-14326
q-$BiVO_4$	Photocatalysis,<br>10 mg catalyst,<br>10 bar CH_4,<br>25 °C	Total: 14 μmol·h^{-1}<br>CH_3OH + HCHO	CH_3OH: 54.8<br>HCHO: 45.2	*Nat. Sustain.* **2021**, 4, 509-515
Au_1/BP	Photocatalysis,<br>200 mg catalyst,<br>30 bar CH_4,<br>90 °C	Total: 11.4 μmol·h^{-1}<br>CH_3OH	CH_3OH: 100	*Nat. Commun.***2021**, 12, 1218
HSiMo/TiO_2	Photocatalysis,<br>20 mg catalyst,<br>30 bar CH_4,<br>150 °C	Total: 28,9 μmol·h^{-1}<br>CH_3OH + HCHO + HCOOH	CH_3OH: 6.3<br>HCHO: 46.6<br>HCOOH: 47.1	*J. Mater. Chem. A* **2021**, 9, 1713-1719
Au-ZSM-5	Thermocataysis,<br>100 mg catalyst,<br>23.2 bar CH_4,<br>240 °C	Total: 1.29 μmol·h^{-1}<br>CH_3OH + CH_3OOH	CH_3OH: 86.0<br>CH_3OOH: 14.0	*Nat. Catal.* **2022**, 5, 45-54
Pd_9Au_1 NW	Thermocatalysis,<br>10 mg catalyst,<br>20.16 bar CH_4,<br>90 °C	Total: 28.9 μmol·h^{-1}<br>CH_3OH + CH_3OOH + HCOOH	CH_3OH: 42.8<br>CH_3OOH: 15.3<br>HCOOH: 41.9	*Appl. Catal. B* **2022**, 308, 121223
Pd-def-In_2O_3	Photocatalysis,<br>20 mg catalyst,<br>19 bar CH_4,<br>25 °C	Total: 60.0 μmol·h^{-1}<br>CH_3OH + CH_3OOH + HCHO	CH_3OH: 2.7<br>CH_3OOH: 14.8<br>HCHO: 82.5	*Nat. Commun.* **2022**, 13, 2930
$Au_{0.2}Cu_{0.15}$/ZnO	Photocatalysis,<br>20 mg catalyst,<br>19 bar CH_4,<br>25 °C	Total: 224.5 μmol·h^{-1}<br>CH_3OH + CH_3OOH + HCHO	CH_3OH: 57.5<br>CH_3OOH: 21.7<br>HCHO: 20.8	*J. Am. Chem. Soc.* **2022**, 144, 740-750

Supplementary Table 2. Reaction conditions, and production rate and selectivity of liquid products reported in Thermo- and Photocatalysis and this work.

**Compound**	**$\Delta_f H_{298}^o$** [kcal mol^{-1}]	**S_{298}^o** [cal mol^{-1} K^{-1}]
H_2	0.0 ± 0.0	31.1 ± 0.1
CH_4	-17.6 ± 0.3	44.4 ± 0.2
H_2O	-58.0 ± 0.2	45.0 ± 0.2
CO	-26.3 ± 0.1	47.1 ± 0.1
CH_3OH	-48.2 ± 0.3	57.2 ± 0.4
O_2	0.0 ± 0.0	48.9 ± 0.1
CO_2	-94.1 ± 0.1	51.0 ± 0.3
HCOOH	-90.5 ± 0.1	59.5 ± 0.5
CH_3OOH	-30.7 ± 0.9	67.1 ± 0.8

Supplementary Table 3. Enthalpy of formation ($\Delta_f H_{298}^o$) and entropy (S_{298}^o) values.
